# Supplementary material for: Trends in hospitalisation for urinary tract infection in adults aged 18–65 by sex in Spain: 2000 to 2015
Source: PLoS One. 2024 Apr 16;19(4):e0298931. doi: 10.1371/journal.pone.0298931 (PMC11020983; doi:10.1371/journal.pone.0298931)
Supplement: S2 File — (DOCX) [file pone.0298931.s002.docx]

|  | **Women** | | | **Men** | | | |
| --- | --- | --- | --- | --- | --- | --- | --- |
| **Year /Age year** | **Pyelonephritis** | **Cystitis** | **Non-specific** | **Pyelonephritis** | **Prostatitis** | **Cystitis** | **Non-specific** |
| **2000** | 40.8 | 0 .9 | 37.7 | 9.7 | 7.7 | 0.3 | 12.5 |
| **2001** | 42.9 | 0.8 | 37.1 | 10.0 | 7.0 | 0.3 | 12.9 |
| **2002** | 44.0 | 0.8 | 35.5 | 9.2 | 7.0 | 0.2 | 13.1 |
| **2003** | 45.1 | 0.7 | 36.7 | 9.1 | 7.1 | 0.2 | 14.1 |
| **2004** | 48.0 | 0.5 | 33.4 | 9.1 | 7.2 | 0.1 | 13.6 |
| **2005** | 48.5 | 0.5 | 30.1 | 9.6 | 7.0 | 0.2 | 13.8 |
| **2006** | 51.2 | 0.4 | 31.0 | 9.1 | 8.0 | 0.2 | 15.2 |
| **2007** | 50.5 | 0.5 | 29.7 | 9.3 | 7.3 | 0.2 | 15.0 |
| **2008** | 51.9 | 0.5 | 29.7 | 9.3 | 7.2 | 0.2 | 14.9 |
| **2009** | 48.7 | 0.4 | 27.9 | 8.4 | 7.6 | 0.2 | 15.7 |
| **2010** | 48.9 | 0.4 | 25.8 | 8.5 | 7.8 | 0.2 | 15.7 |
| **2011** | 48.7 | 0.4 | 24.1 | 8.6 | 9.5 | 0.3 | 16.5 |
| **2012** | 50.0 | 0.4 | 24.1 | 8.3 | 9.1 | 0.2 | 16.5 |
| **2013** | 53.3 | 0.4 | 24.8 | 9 .0 | 10.2 | 0.3 | 18.4 |
| **2014** | 55.3 | 0.5 | 24.9 | 9.2 | 10.6 | 0.3 | 19.3 |
| **2015** | 55.5 | 0.4 | 25.8 | 9.5 | 11.7 | 0.3 | 19.5 |

**Supplement 2. Type of Urinary Tract Infections hospital admissions rates per 100,000 inhabitants by sex, Spain, 2000-2015**
