# Supplementary material for: Trends in hospitalisation for urinary tract infection in adults aged 18–65 by sex in Spain: 2000 to 2015
Source: PLoS One. 2024 Apr 16;19(4):e0298931. doi: 10.1371/journal.pone.0298931 (PMC11020983; doi:10.1371/journal.pone.0298931)
Supplement: S3 File — (DOCX) [file pone.0298931.s003.docx]

**Supplement 3. Trends in hospitalizations rates due to Cystitis**

**A. Women**


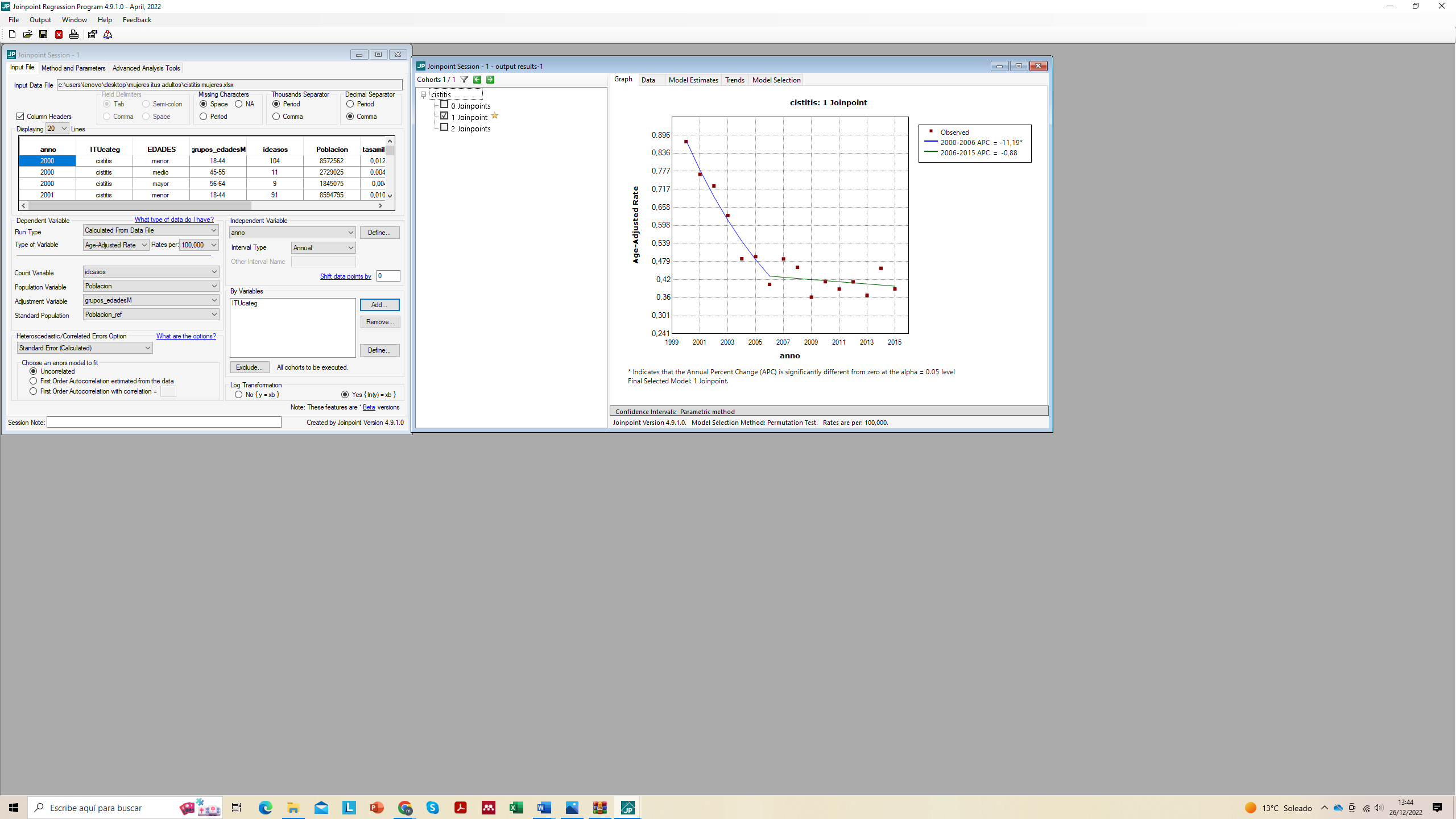


**B. Men**


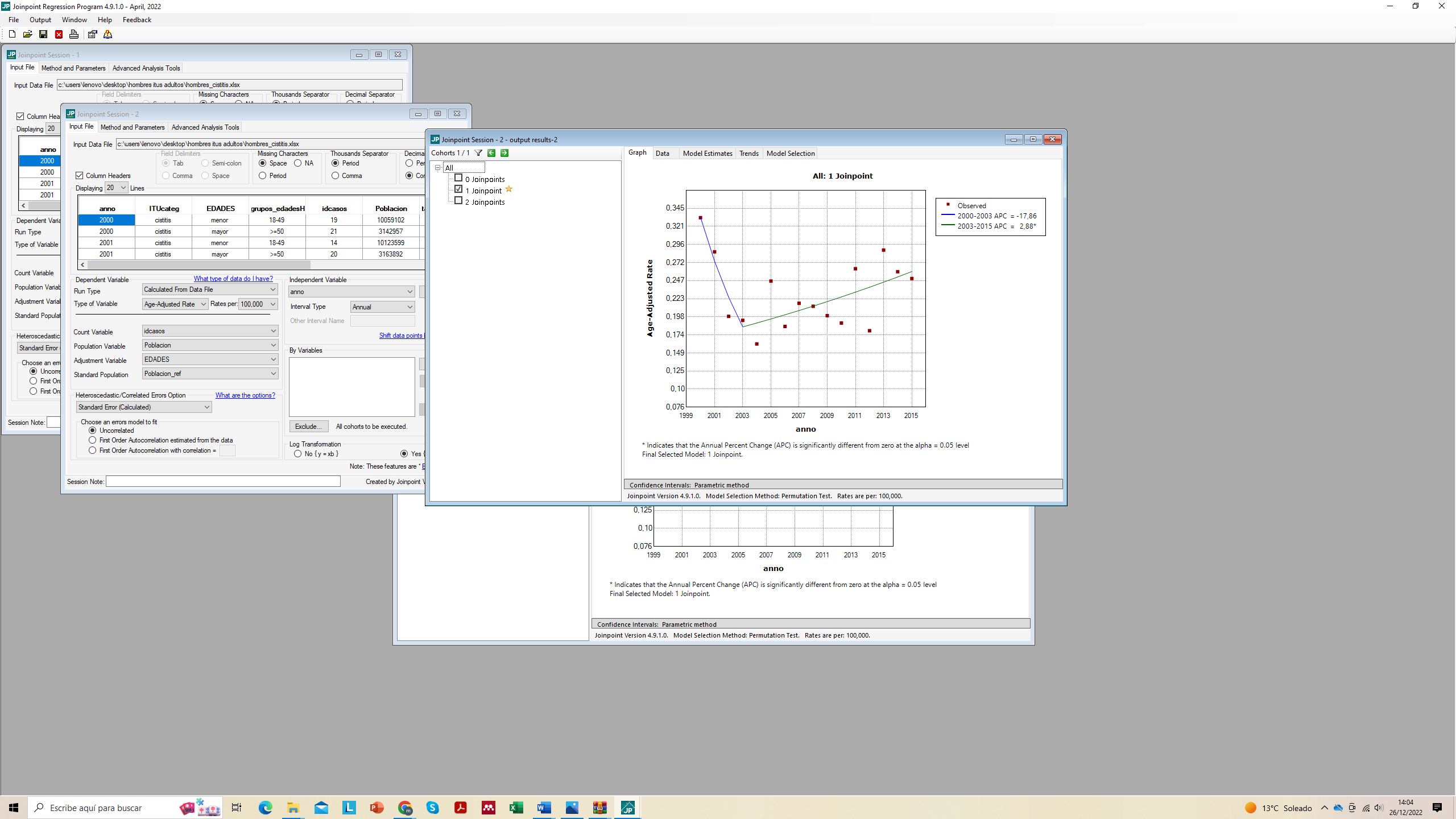


|  | Women (A) | Men (B) |
| --- | --- | --- |
| **APPC** | −5.1 | −1.7 |
| **(CI 95%)** | (−7.0 to −3.2) | (−6.1 to 3.1) |
| **APC** | 11.2 | -17.9 |
| **(CI 95%)** | (−14.7 to −7.6) | (-35.3 to 4.2) |
| **Years** | 2000-2006 | 2000-2003 |
|  | -0.9 | 2.9 |
|  | (-3.4 to 1.7) | (0.1 to 5.8) |
|  | 2006-2015 | 2003-2015 |
| APC= annual percent change, AAPC average annual percent change | | |
